# Supplementary material for: The head mesodermal cell couples FMRFamide neuropeptide signaling with rhythmic muscle contraction in C. elegans
Source: Nat Commun. 2023 Jul 14;14:4218. doi: 10.1038/s41467-023-39955-8 (PMC10349088; doi:10.1038/s41467-023-39955-8)
Supplement: Supplementary file 1 — Supplementary Information [file 41467_2023_39955_MOESM1_ESM.pdf]

The head mesodermal cell couples FMRFamide neuropeptide signaling with muscle contraction during a rhythmic behavior in *C. elegans*

Ukjin Choi, Mingxi Hu, Qixin Zhang, and Derek Sieburth

Derek Sieburth
Email: sieburth@usc.edu

Supplementary Information:

Supplementary Figure 1 to Figure 8
Supplementary Table 1 to Table 2
Supplementary References

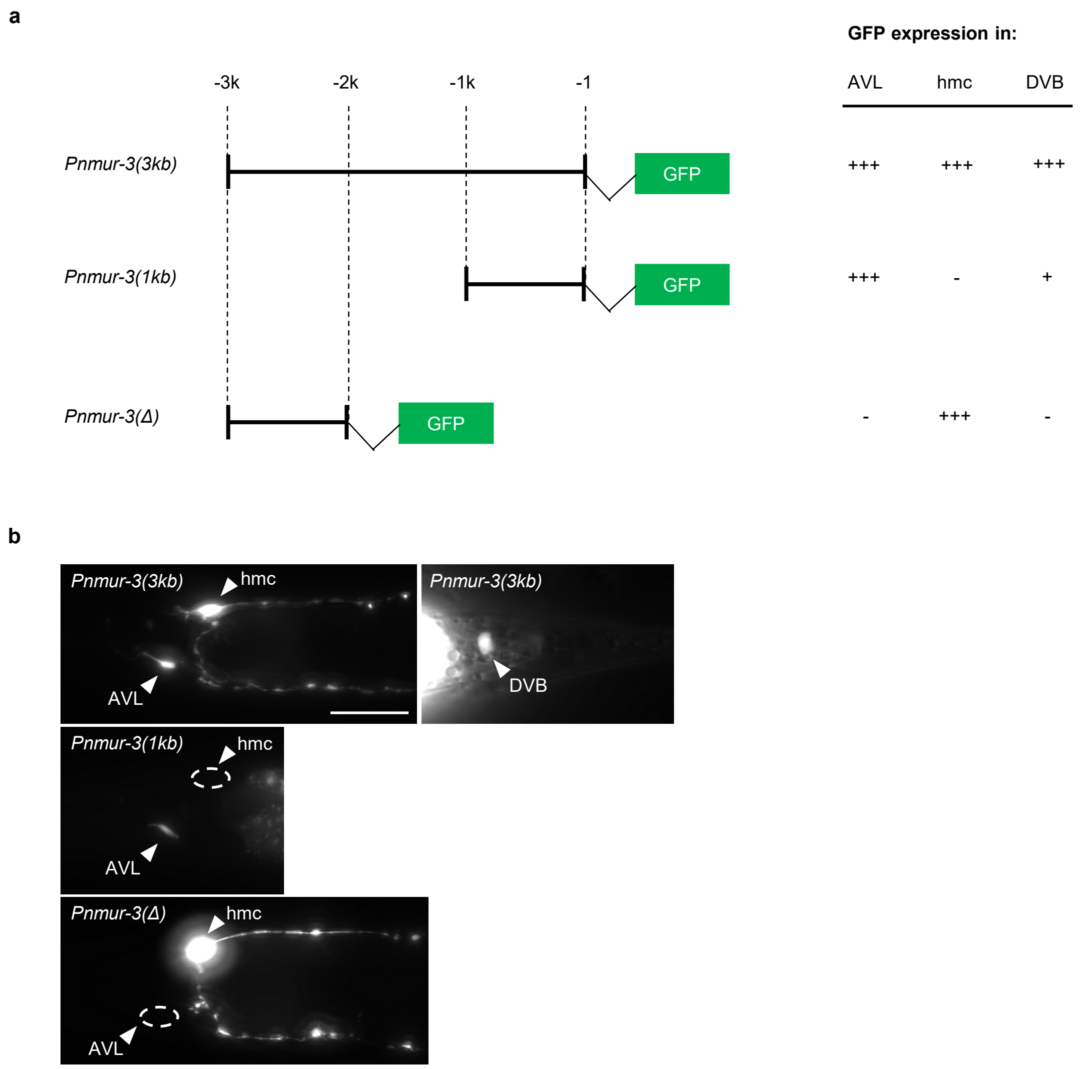

Supplementary Figure 1: Analysis of the expression patterns of *nmur-3* promoter fragments. a Schematic of the *nmur-3* promoter. The *nmur-3(3kb)* promoter fragment extends from -1bp to -2956bp relative to the ATG codon of *nmur-3*, and drives expression of GFP in AVL, DVB and hmc. The P*nmur-3(1kb)* promoter fragment extends from -1bp to -1053 bp relative to the ATG codon of *nmur-3,* and drives GFP expression in AVL but not in hmc. The P*nmur-3(Δ)* promoter fragment extends from -2026 bp to -2956 bp and drives GFP expression primarily in hmc but not in AVL. +++ indicates that 80-100% of animals exhibited fluorescence in the indicated cell, + indicates that <10% of animals exhibited fluorescence in the indicated cell, and – indicates that 0% of animals exhibited fluorescence in the indicated cell. At least 20 animals were examined for each transgenic line. **b** Representative images of adults from the transgenic lines described in **a**. Scale bar, 40 μm.

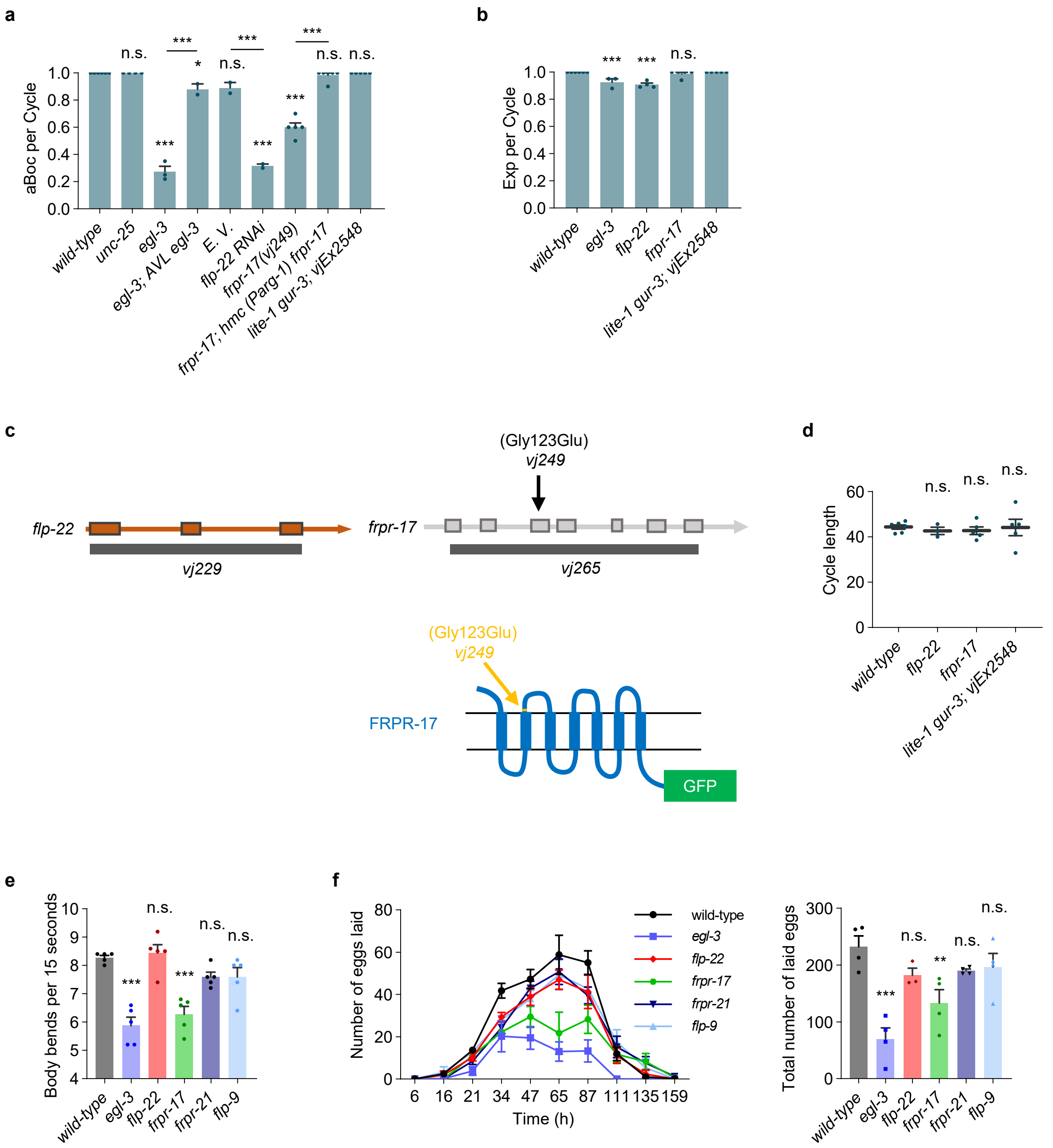

Supplementary Figure 2: Phenotypic analysis of *flp-22* and *frpr-17* mutants. a Quantification of the number of aBocs per cycle in adult animals of the indicated genotypes. "*AVL egl-3*" denotes *egl-3* cDNA expressed in AVL using the *nmur-3(1kb)* promoter. "*hmc (Parg-1) frpr-17*" denotes *frpr-17* cDNA expressed in hmc using the *arg-1* promoter. *vjEx2548* is the GCaMP imaging strains used in this study. Data are presented as mean values ± SEM. *n* = 6, 4, 3, 2, 2, 2, 5, 6, 5 independent animals. *** P<0.001 and * P<0.05 in one-way ANOVA with Bonferroni's correction for multiple comparisons; n.s., not significant. **b** Quantification of expulsion (Exp) frequency in the indicated genotypes. Data are presented as mean values ± SEM. *n* = 6, 3, 4, 5, 5 independent animals. *** P<0.001 in one-way ANOVA with Dunnett's correction for multiple comparisons; n.s., not significant. **c** *Top, left:* genomic organization of the *flp-22* locus showing the location and lesion of *vj229* allele. *Top, right:* genomic organization of the *frpr-17* locus showing the locations and the lesions of the *vj249* and *vj265* alleles. *vj249* is a glycine to glutamic acid substitution in exon 3. *Bottom:* diagram showing the predicted structure of the FRPR-17 protein and the position of the amino acid substitution in the extracellular domain in the *vj249* mutant. **d** Quantification of cycle lengths in the indicated genotypes. Data are presented as mean values ± SEM. *n* = 6, 3, 5, 5 independent animals. One-way ANOVA with Dunnett's correction for multiple comparisons; n.s., not significant. **e** Locomotion, measured by body bends per minute, of young adult animals of the indicated genotypes. Data are presented as mean values ± SEM. *n* = 5 independent animals. *** P<0.001 in one-way ANOVA with Dunnett's correction for multiple comparisons; n.s., not significant. **f** Egg laying rates and total number of eggs laid in the indicated genotypes. Data are presented as mean values ± SEM. *n* = 4, 4, 3, 4, 4, 4 independent animals. *** P<0.001 and ** P<0.01 one-way ANOVA with Dunnett's correction for multiple comparisons; n.s., not significant.

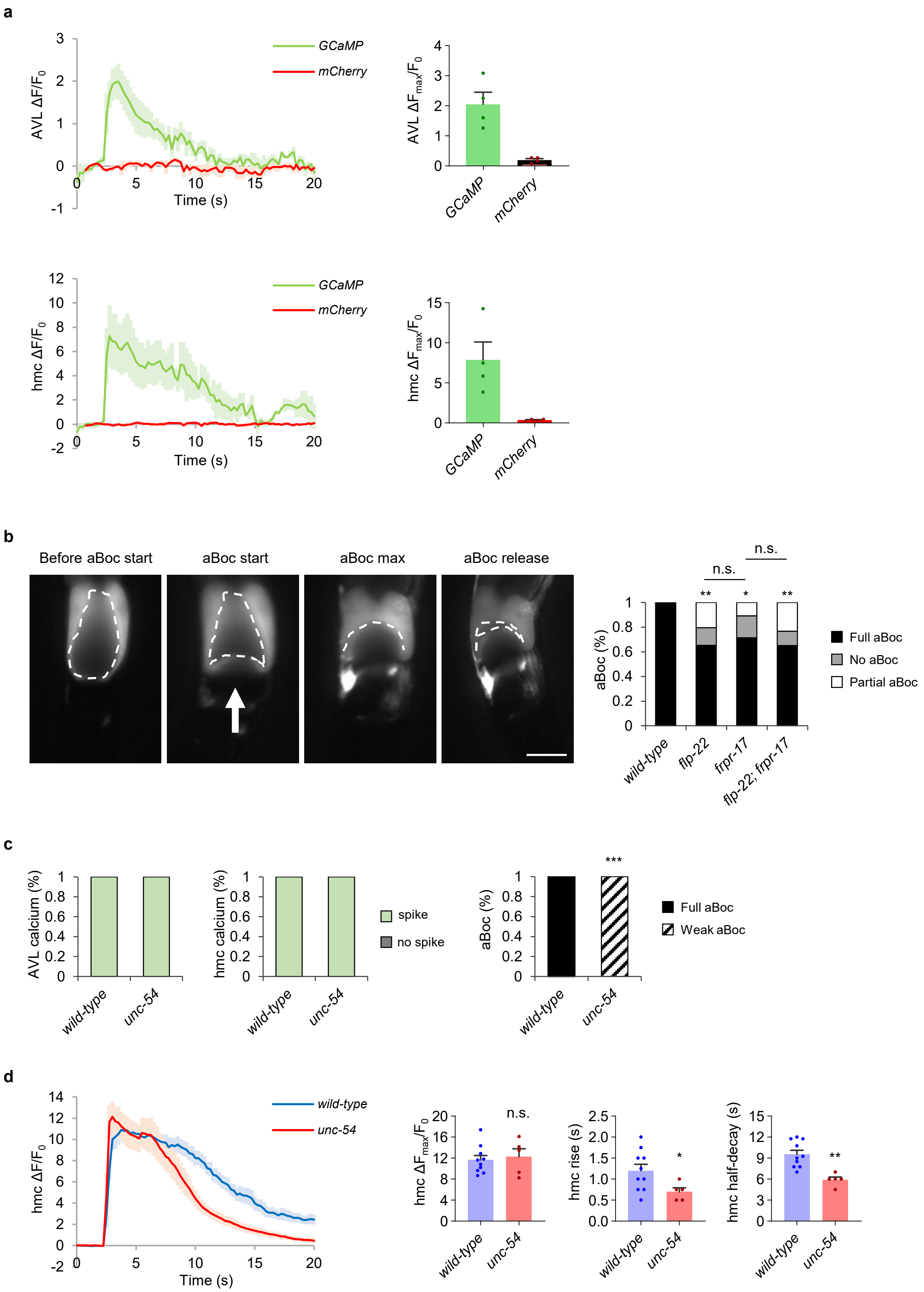

a
GCaMP
mCherry
AVL ΔF/F0
Time (s)
AVL ΔFmax/F0
hmc ΔF/F0
hmc ΔFmax/F0
b
Before aBoc start
aBoc start
aBoc max
aBoc release
n.s.
n.s.
**
*
**
aBoc (%)
Full aBoc
No aBoc
Partial aBoc
wild-type
flp-22
frpr-17
flp-22; frpr-17
c
AVL calcium (%)
hmc calcium (%)
spike
no spike
aBoc (%)
***
Full aBoc
Weak aBoc
wild-type
unc-54
d
wild-type
unc-54
hmc ΔF/F0
Time (s)
hmc ΔFmax/F0
n.s.
hmc rise (s)
*
hmc half-decay (s)
**

Supplementary Figure 3: Movement has minimal impact on GCaMP fluorescence intensity, aBoc can be detected using time lapse images, and muscles do not activate hmc. a *Left:* average traces of fluorescence intensity in AVL or hmc in animals co-expressing GCaMP and mCherry. Each trace is aligned to the calcium spike initiation time, and the solid lines indicate average fold change in fluorescence intensity and the shades indicate SEM. *Right:* quantification of the average peak amplitude of CGaMP and mCherry. Data are presented as mean values ± SEM. *n* = 4 cycles from different animals. **b** *Left:* representative images from the live calcium imaging showing contractions of the neck muscle. Posterior is up and dorsal is to the left in these images. The lumen of the anterior intestine is outlined with a white dotted line. The lumen of the intestine begins to displace posteriorly at the initiation of aBoc (arrow) and reaches maximum displacement after about one second before beginning to relax. Similar expression patterns were observed in all 10 animals examined. Scale bar, 40 μm. *Right:* in wild-type animals, the duration of the maximal aBoc contraction is at least 500 ms (full aBoc). In the indicated mutants, the maximal displacement is not reached or it is less than 500 ms in about 30% of cycles (partial and no aBoc). ** P<0.01 and * P<0.05 in two-sided chi-square test with Bonferroni's correction for multiple comparisons; n.s., not significant. **c** Calcium spike frequency in AVL and hmc and aBoc frequency in *unc-54*/myosin mutants from time lapse imaging. "Weak aBoc" denotes a contraction with a very little movement to due to compromised muscle function. Wild-type: 33 cycles in 8 animals, *unc-54*: 10 cycles in 3 animals *** P<0.001 in two-sided Fisher's exact test. **d** *Left:* average traces of calcium dynamics in hmc in the indicated genotypes. Each trace is aligned to the calcium spike initiation time and the solid lines indicate average fold change in fluorescence intensity and the shades indicate SEM. *Right:* quantification of the average peak amplitude, rise time, and half-decay time of CGaMP. Data are presented as mean values ± SEM. *n* = 10, 5 cycles from different animals. ** P<0.01 and * P<0.05 in two-tailed Student's t-test; n.s., not significant.

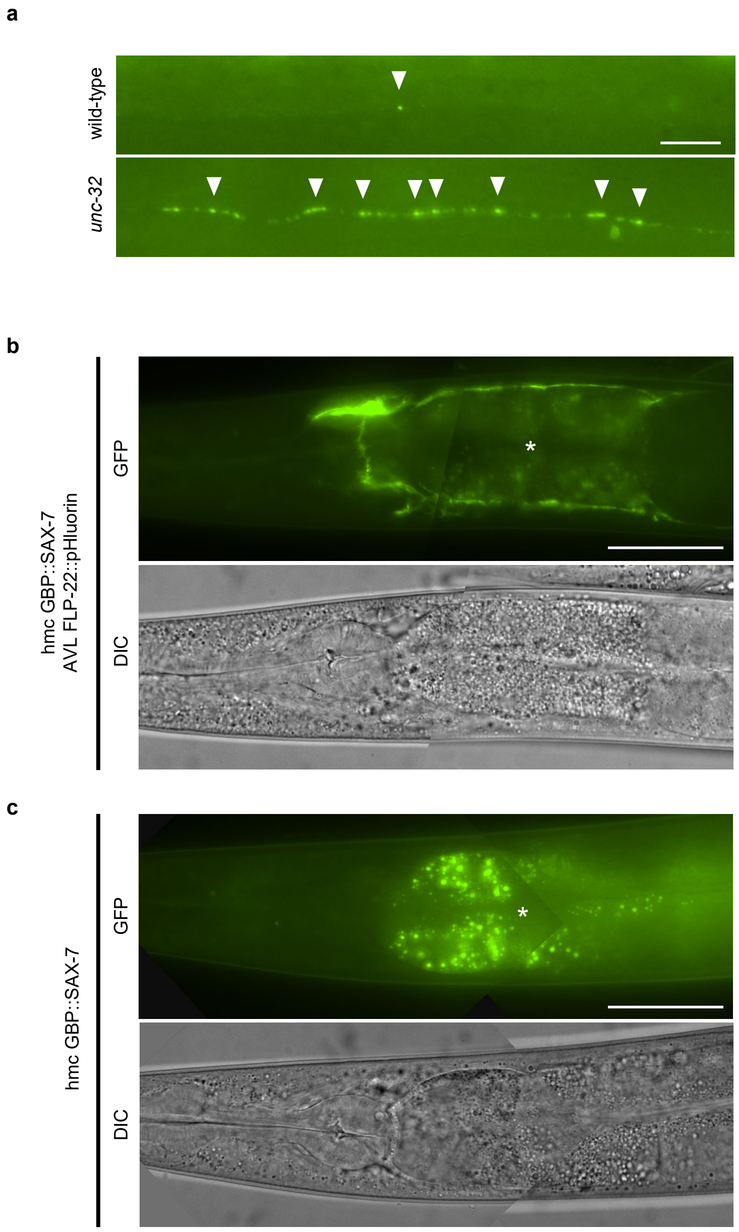

Supplementary Figure 4: FLP-22 secreted from AVL can reach hmc. a Representative image of the proximal AVL axon of adults expressing FLP-22::pHluorin in AVL in the indicated genotypes. Arrowheads denote clusters of DCVs visible in the proximal axon containing unquenched FLP-22::pHluorin. Similar expression patterns were observed in all 25 animals examined for each genotype. Scale bar, 20 µm. **b** Representative images of adults co-expressing FLP-22::pHluorin in AVL and GBP::SAX-7 in hmc. Fluorescence is observed on the surface of hmc, indicating that FLP-22::pHluorin was secreted from AVL and is associated with hmc through interaction with the GFP binding domain of GBP::SAX-7. Similar expression patterns were observed in all 15 animals examined. Scale bar, 40 µm. **c** Representative images showing adults expressing only GBP::SAX-7 in hmc. Similar expression patterns were observed in all 15 animals examined. Scale bar, 40 µm.

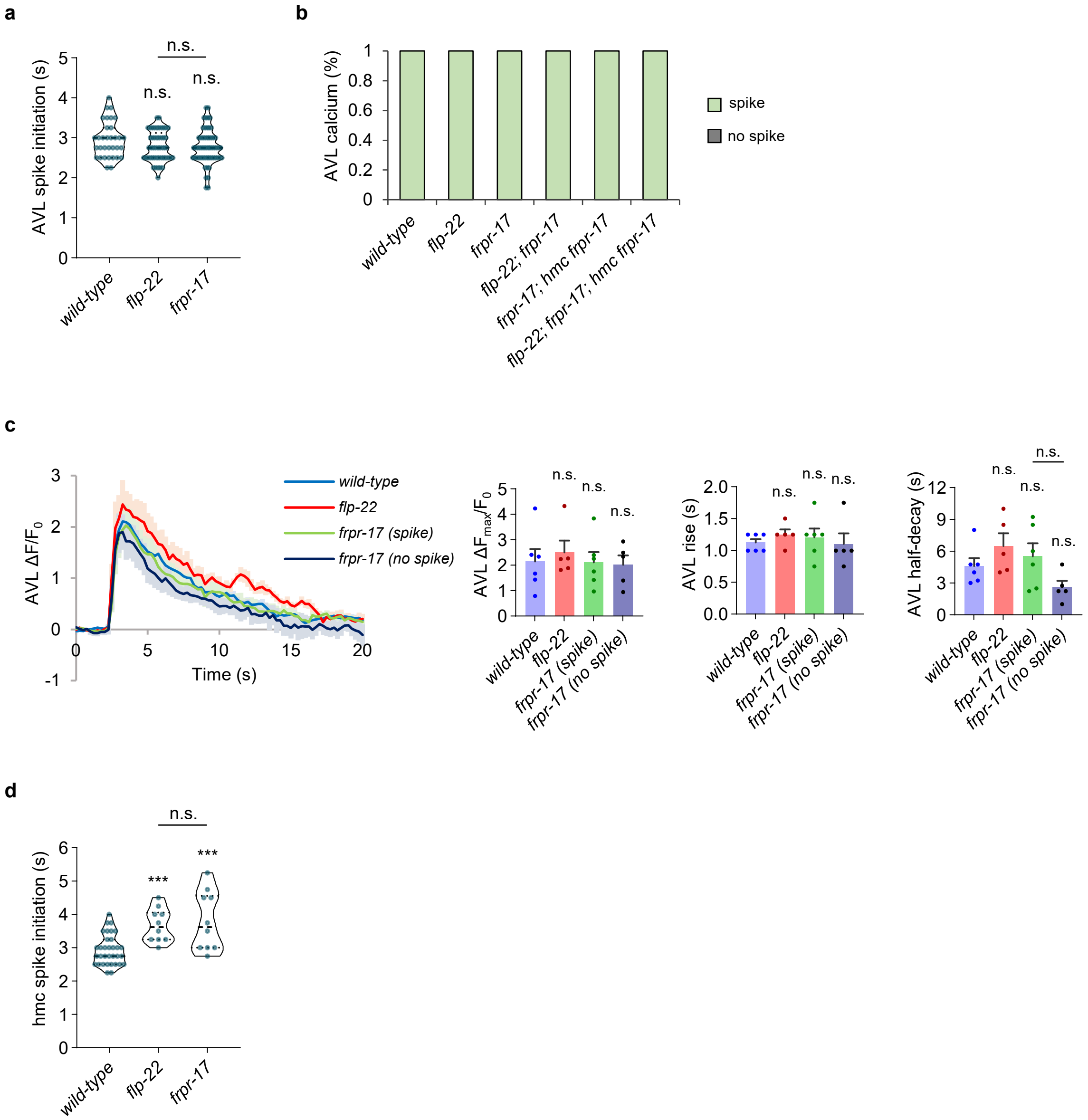

Supplementary Figure 5: *flp-22* and *frpr-17* mutants do not alter calcium dynamics in AVL. a Violin plots of calcium spike initiation time in hmc after the end of intestinal calcium oscillation. Dashed line refers median and dotted lines refer quartiles. One-way ANOVA with Bonferroni's correction for multiple comparisons; n.s., not significant. **b** Quantification of the number of calcium spikes observed in AVL during DMP in adult animals of the indicated genotypes. "*hmc frpr-17*" denotes expressing *frpr-17* cDNA under the *nmur-3(Δ)* promoter. For a, b, and d, wild-type: 33 cycles in 8 animals, *flp-22*: 49 cycles in 9 animals, *frpr-17*: 56 cycles in 13 animals, *flp-22; frpr-17*: 43 cycles in 9 animals, *frpr-17; hmc frpr-17*: 20 cycles in 7 animals, *flp-22; frpr-17; hmc frpr-17*: 42 cycles in 9 animals. **c** *Left:* traces of calcium dynamics in AVL aligned to the calcium spike initiation time. Solid lines indicate average fold change in GCaMP intensity and shades indicate SEM. *Right:* quantification of average peak amplitudes, rise times and decay times from the traces on the right. Data are presented as mean values ± SEM. n = 6, 5, 6, 5 cycles from different animals. One-way ANOVA with Bonferroni's correction for multiple comparisons; n.s., not significant. **d** Violin plots of calcium spike initiation time in hmc after the end of intestinal calcium oscillation. Dashed line refers median and dotted lines refer quartiles. *** P<0.001 in one-way ANOVA with Bonferroni's correction for multiple comparisons; n.s., not significant.

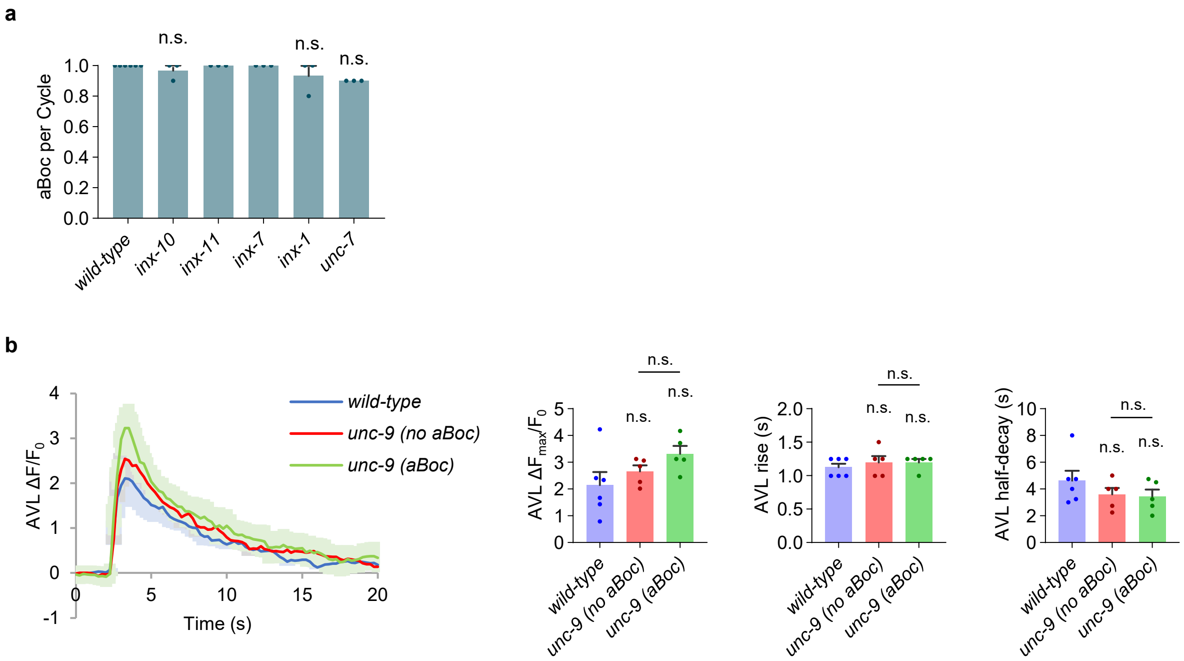

Supplementary Figure 6: aBoc frequency of innexin mutants and calcium dynamics in *unc-9* mutants. a aBoc frequencies of the indicated genotypes. Data are presented as mean values ± SEM. *n* = 6, 3, 3, 3, 3, 3 independent animals. One-way ANOVA with Dunnett's correction for multiple comparisons; n.s., not significant. **b** *Left:* average traces of calcium dynamics in AVL or hmc aligned to the calcium spike initiation time in the indicated mutants. Solid lines indicate average fold change in GCaMP intensity and shades indicate SEM. *unc-9 (no aBoc)* refers to cycles with calcium spike followed by no aBoc and *unc-9 (aBoc)* refers to cycles with calcium spike followed by aBoc. *Right:* quantification of the average peak amplitude, rise time, and half-decay time. Data are presented as mean values ± SEM. *n* = 6, 5, 5 cycles from different animals. One-way ANOVA with Bonferroni's correction for multiple comparisons; n.s., not significant.

a

GPCR	hmc mean counts
frpr-17	95949
frpr-21	29742
nmur-3	23802
srd-32	11686
npr-23	6707
T11F9.1	5145
frpr-4	4702

b

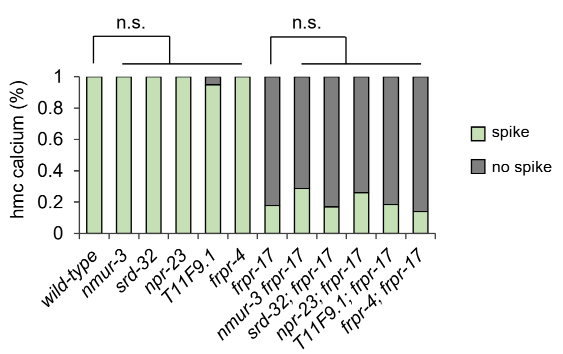

c

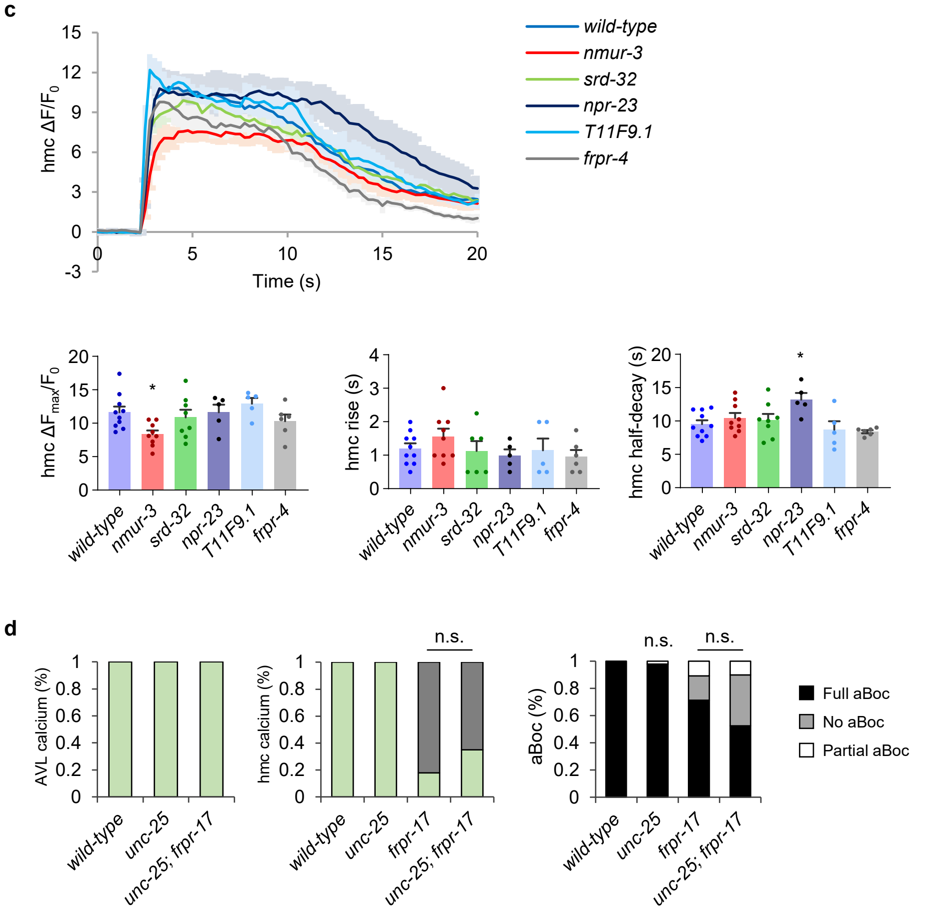

Supplementary Figure 7: aBoc frequency and calcium dynamics of receptors highly expressed in hmc. a Table of the GPCRs highly expressed in hmc and their relative abundance (adapted from [1]). **b** Quantification of the number of calcium spikes observed in hmc in each cycle in adult animals of the indicated genotypes. Wild-type: 33 cycles in 8 animals, *nmur-3*: 21 cycles in 7 animals *srd-32*: 25 cycles in 9 animals, *npr-23*: 23 cycles in 6 animals, *T11F9.1*: 20 cycles in 9 animals, *frpr-17*: 56 cycles in 13 animals, *nmur-3 frpr-17*: 49 cycles in 9 animals, *srd-32; frpr-17*: 47 cycles in 10 animals, *npr-32; frpr-17*: 27 cycles in 7 animals, *T11F9.1; frpr-17*: 49 cycles in 9 animals, *frpr-4; frpr-17*: 43 cycles in 9 animals. Two-sided chi-square test with Bonferroni's correction for multiple comparisons; n.s., not significant. **c** *Above:* average traces of calcium dynamics in hmc aligned to the calcium spike initiation time. Solid lines indicate average fold change in GCaMP intensity and shades indicate standard errors. *Below:* quantification of the average peak amplitude, rise time, and half-decay time. Data are presented as mean values ± SEM. *n* = 10, 9, 8, 5, 5, 6 cycles from different animals. * P<0.05 in one-way ANOVA with Dunnett's correction for multiple comparisons. **d** Quantification of the frequency of calcium spikes observed in AVL and hmc and aBocs from time lapse images of the indicated genotypes. Wild-type: 33 cycles in 8 animals, *unc-25*: 46 cycles in 7 animals *unc-25; frpr-17*: 40 cycles in 6 animals. Two-sided chi-square test with Bonferroni's correction for multiple comparisons; n.s., not significant.

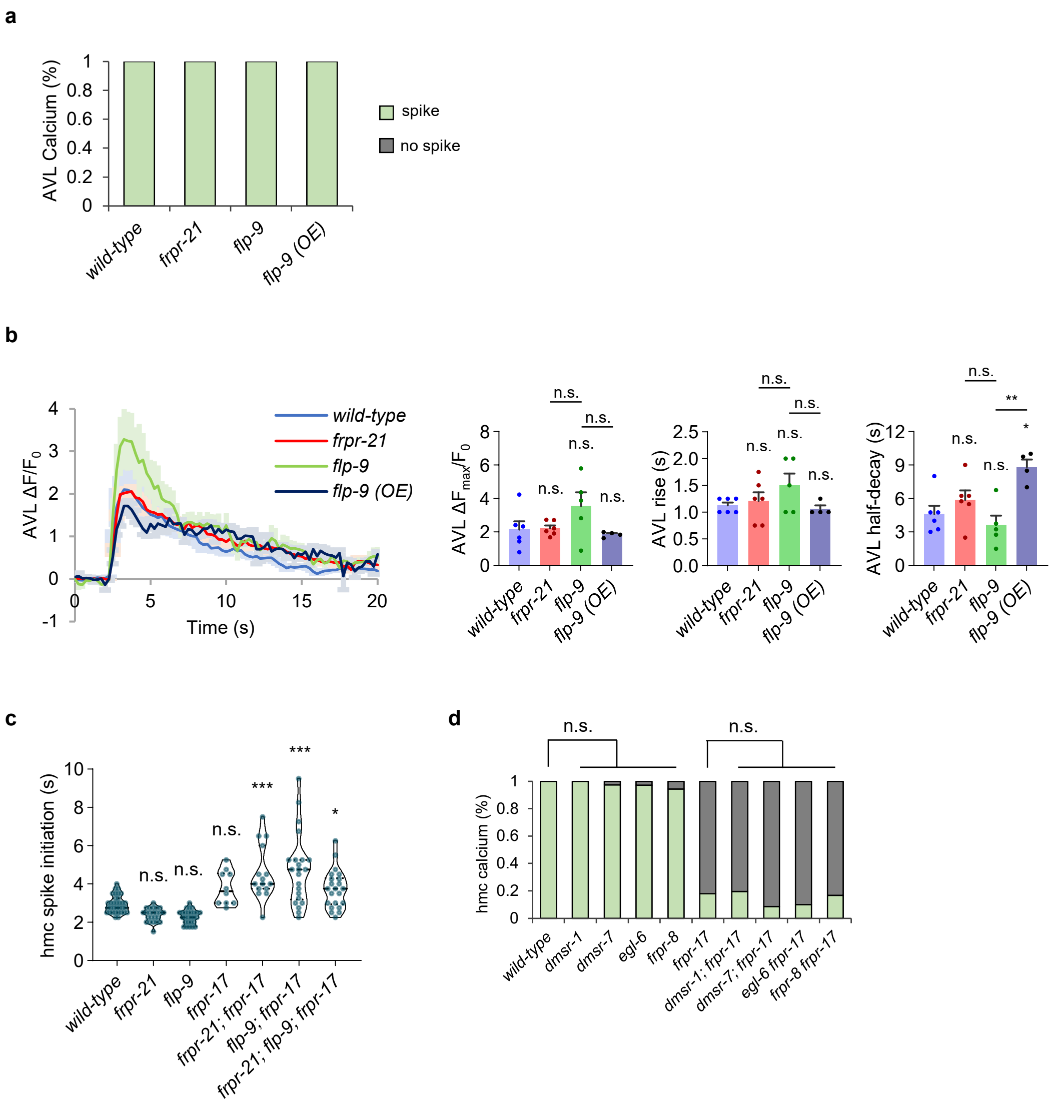

Supplementary Figure 8: Calcium dynamics in *flp-9* mutants and putative *flp-9* GPCR mutants. a Quantification of the number of calcium spikes observed in AVL in each cycle in adult animals of the indicated genotypes. Wild-type: 33 cycles in 8 animals, *frpr-21*: 23 cycles in 9 animals, *flp-9*: 27 cycles in 8 animals, *flp-9 (OE)*: 27 cycles in 5 animals. **b** *Left:* average traces of calcium dynamics in AVL aligned to the calcium spike initiation time in the indicated mutants. The solid lines indicate average fold change in GCaMP intensity and the shades indicate SEM. *Right:* quantification of the average peak amplitude, rise time, and half-decay time. Data are presented as mean values ± SEM. *n* = 6, 6, 5, 4 cycles from different animals. * P<0.05 in one-way ANOVA with Bonferroni's correction for multiple comparisons; n.s., not significant. **c** Violin plots of calcium spike initiation time in hmc after the end of intestinal calcium oscillation. Dashed line refers median and dotted lines refer quartiles. *** P<0.001 and ** P<0.01 in one-way ANOVA with Dunnett's correction for multiple comparisons; n.s., not significant. **d** Quantification of the number of calcium spikes observed in hmc in each cycle in adult animals of the indicated genotypes. Wild-type: 33 cycles in 8 animals, *dmsr-1*: 30 cycles in 5 animals, *dmsr-7*: 36 cycles in 5 animals, *egl-6*: 34 cycles in 5 animals, *frpr-8*: 35 cycles in 6 animals, *frpr-17*: 56 cycles in 13 animals, *dmsr-1; frpr-17*: 41 cycles in 7 animals, *dmsr-7; frpr-17*: 47 cycles in 7 animals, *egl-6 frpr-17*: 40 cycles in 7 animals, *frpr-8 frpr-17*: 24 cycles in 7 animals. Two-sided chi-square test with Bonferroni's correction for multiple comparisons; n.s., not significant.

Supplementary Table 1: Strains, transgenic lines, and plasmids used in this study

Strain	Genotype	Figures	Source
N2	Wild-type Bristol strain	Fig. 1, Fig. S2a, b, d, e, f, Fig. 3a, Fig. 4b, Fig. S5A, Fig. 5A, D	
OJ794	*nlp-40(tm4085) I*	Fig. 1b	[2]
OJ6846	*aex-2(vj304) X*	Fig. 1b	This paper
OJ3271	*flp-22(vj229) I*	Fig. 1c, Fig. S2b, d, e, f Fig. 5a	This paper
OJ6057	*frpr-17(vj265) X*	Fig. 1c, Fig. S2b, d, e, f	This paper
	flp-22(vj229) I; frpr-17(vj265) X	Fig. 1c	This paper
OJ6754	*hlh-8(nr2061) X*	Fig. 1d	This paper
OJ7173	*flp-22(vj229) I; hlh-8(nr2061) X*	Fig. 1d	This paper
OJ1218	*unc-25(e156) III*	Fig. S2a	This paper
OJ2424	*egl-3(nr2090) V*	Fig. S2a, b, e, f	This paper
OJ5308	*frpr-17(vj249) X*	Fig. S2a	This paper
OJ3584	*egl-30(ad806) I*	Fig. 3a	This paper
OJ8203	*unc-9(e101) X*	Fig. 4b	This paper
OJ2451	*inx-10(ok2714) V*	Fig. S6a	This paper
OJ4154	*inx-11(ok2783) V*	Fig. S5a	This paper
OJ9407	*inx-7(ok2319) IV*	Fig. S5a	This paper
OJ2446	*inx-1(tm3524) X*	Fig. S5a	[3]
OJ3650	*unc-7(e5) X*	Fig. S5a	This paper
OJ7399	*frpr-21(tm4669) II*	Fig. 5a, Fig. S2e, f	This paper
OJ6524	*flp-22(vj229) I; frpr-21(tm4669) II*	Fig. 5a	This paper
OJ3844	*flp-9(yn36) IV*	Fig. 5e, Fig. S2e, f	This paper
OJ7009	*vjEx2445 [pMH569 (Pnmur-3(1kb)::ICE), 10 ng/μL]*	Fig. 1b	This paper
OJ1626	*nlp-40(tm4085) I; vjEx368 [pHW61 (Pges-1::nlp-40 cDNA), 25 ng/μL]*	Fig. 1b	[2]
OJ9177	*aex-2(vj304) X; vjEx3004 [pDS728 (Pnmur-3(1kb)::aex-2 cDNA), 10 ng/μL]*	Fig. 1b	This paper
OJ7011	*vjEx2447 [pDS729 (Pnmur-3(1kb)::TeTx), 10 ng/μL]*	Fig. 1b	This paper
OJ9314	*flp-22(vj229) I; vjEx1837 [pHM363 (Punc-47::flp-22 cDNA), 25 ng/μL]*	Fig. 1c	This paper
OJ5082	*flp-22(vj229) I; vjEx1534 [pHM184 (Punc-129::flp-22 cDNA), 25 ng/μL]*	Fig. 1c	This paper
OJ7439	*flp-22(vj229) I; vjEx2466 [pDS747 (Pnmur-3(1kb)::flp-22 cDNA), 10 ng/μL]*	Fig. 1c	This paper
OJ9395	*frpr-17(vj249) X; vjEx1596 [pDS573 (Prab-3::frpr-17 cDNA), 5 ng/μL]*	Fig. 1c	This paper
OJ5372	*frpr-17(vj249) X; vjEx1580 [pDS567 (Pmyo-3::frpr-17 cDNA) 10 ng/μL]*	Fig. 1c	This paper
OJ9396	*frpr-17(vj265) X; vjEx2830 [pUC259 (Pnmur-3(Δ)::frpr-17 cDNA) 25 ng/μL]*	Fig. 1c	This paper

OJ6607	*vjEx2174 [pUC168 (Pnmur-3::GFP) 50 ng/µL]*	Fig. 1d, Fig. S1	This paper
OJ7659	*vjEx2653 [pUC249 (Pnmur-3(Δ)::ICE), 25 ng/µL]*	Fig. 1d	This paper
OJ7716	*frpr-17(vj265) X; vjEx2653 [pUC249 (Pnmur-3(Δ)::ICE), 25 ng/µL]*	Fig. 1d	This paper
OJ7656	*vjEx2651 [pUC246 (Pnmur-3(Δ)::PH domain::miniSOG::SL2::mCherry), 25 ng/µL]*	Fig. 1d	This paper
OJ6889	*otIs348; vjEx2349 [pUC214 (Pnmur-3(1kb)::GFP) 2 ng/µL]*	Fig. S1	This paper
OJ7018	*vjEx2454 [pUC218 (Pnmur-3(Δ)::GFP) 50 ng/µL]*	Fig. S1	This paper
OJ7149	*egl-3(nr2090) V; vjEx2535 [pMH576 (Pnmur-3(1kb)::egl-3::Venus) 5 ng/µL]*	Fig. S2a	This paper
OJ6220	*frpr-17(vj265); vjEx1926 [pUC163 (Parg-1::Pegl-18::frpr-17 cDNA::mCherry) 50 ng/µL]*	Fig. S2a	This paper
OJ8149	*lite-1(ce314) gur-3(ok2245) X; vjEx2548 [pUC191 (Pnmur-3(3kb)::GCaMP6) 12 ng/µL + pHW107 (Pnlp-40::GCaMP3) 10 ng/µL]*	Fig. 2, Fig. S2b, d, Fig. S3b-d, Fig. S5, Fig. 3b-e, Fig. 4d-f, Fig. S6b, Fig. 5b, c, f, Fig. S7b-d, Fig. S8	This paper
OJ9089	*lite-1(ce314) gur-3(ok2245) X; vjEx2548; vjEx2957 [pMH569 (Pnmur-3(1kb)::ICE) 10 ng/µL]*	Fig. 2d	This paper
OJ9397	*lite-1(ce314) gur-3(ok2245) aex-2(vj302) X; vjEx2548*	Fig. 2d, e	This paper
OJ9398	*lite-1(ce314) gur-3(ok2245) aex-2(vj302) X; vjEx2548; vjEx2985 [pDS728 (Pnmur-3(1kb)::aex-2 cDNA) 7 ng/µL]*	Fig. 2d, e	This paper
OJ8231	*flp-22(vj229) I; lite-1(ce314) gur-3(ok2245) X; vjEx2548*	Fig. 2f, g, Fig. S3b, Fig. S5	This paper
OJ9634	*flp-22(vj229) I; lite-1(ce314) gur-3(ok2245) X; vjEx2548; vjEx3125 [pDS747 (Pnmur-3(1kb)::flp-22 cDNA) 10 ng/µL]*	Fig. 2f	This paper
OJ8229	*lite-1(ce314) gur-3(ok2245) frpr-17(vj265) X; vjEx2548*	Fig. 2f, g, Fig. S3b, Fig. S5, Fig. 3b, c, e, Fig. 5b, c, Fig. S7b, d, Fig. S8c, d	This paper
OJ9399	*flp-22(vj229) I; lite-1(ce314) gur-3(ok2245) frpr-17(vj265) X; vjEx2548*	Fig. 2f, Fig. S3b, Fig. S5b	This paper
OJ8725	*lite-1(ce314) gur-3(ok2245) frpr-17(vj265) X; vjEx2548; vjEx2830 [pUC259 (Pnmur-3(Δ)::frpr-17 cDNA) 25 ng/µL]*	Fig. 2f, Fig. S5b	This paper
OJ8783	*flp-22(vj229) I; lite-1(ce314) gur-3(ok2245) frpr-17(vj265) X; vjEx2548; vjEx2830 [pUC259 (Pnmur-3(Δ)::frpr-17 cDNA) 25 ng/µL]*	Fig. 2f, Fig. S5b	This paper
OJ9721	*lite-1(ce314) gur-3(ok2245) X; vjEx3153 [pUC297 (Pnmur-3(Δ)::GCaMP6::SL2::mCherry) 12 ng/µL]*	Fig. S3a	This paper
OJ9711	*unc-54(e1108) I; vjEx2548*	Fig. S3c, d	This paper
OJ7370	*vjEx2589 [pDS788 (Pnmur-3(1kb)::flp-22::pHluorin::flp-22)*	Fig. S4a	This paper
OJ8077	*unc-32(e189) III; vjEx2589 [pDS788 (Pnmur-3(1kb)::flp-22::pHluorin::flp-22)*	Fig. S4a	This paper
OJ9320	*vjEx2589 [pDS788 (Pnmur-3(1kb)::flp-22::pHluorin::flp-22) 5 ng/µL]; vjEx3038 [pDS834 (Pnmur-3(Δ)::GBP::sax-7) 20 ng/µL]*	Fig. S4b, c	This paper
OJ8127	*vjEx2749 [pUC247 (Pnmur-3(Δ)::kin-2a(G310D)) 50 ng/µL]*	Fig. 4a	This paper

OJ9150	*lite-1(ce314) gur-3(ok2245) X; vjEx2548; vjEx2987 [pUC247 (Pnmur-3(Δ)::kin-2a(G310D)) 50 ng/μL]*	Fig. 3b-e	This paper
OJ8824	*lite-1(ce314) gur-3(ok2245) frpr-17(vj265) X; vjEx2548; vjEx2871 [pUC273 (Pnmur-3(Δ)::kin-1a(H96Q,W205R)) 25 ng/μL]*	Fig. 3b-e	This paper
OJ9714	*unc-9(e101) X; vjEx3150 [pUC291 (Pnmur-3(Δ)::unc-9 cDNA::mTurquoise2) 25 ng/μL]*	Fig. 4b	This paper
OJ9148	*unc-9(e101) X; vjEx3000 [pUC258 (Pmyo-3::unc-9 cDNA) 10 ng/μL]*	Fig. 4b	This paper
OJ9356	*vjEx3049 [pUC265 (Pnmur-3(Δ)::frpr-17 cDNA::Venus) 20 ng/μL + pUC291 (Pnmur-3(Δ)::unc-9 cDNA::linker::mTurquoise2) 20 ng/μL + pDS833 (Pnmur-3(Δ)::wdr-23b NLS::mCherry) 20 ng/μL]*	Fig. 4c	This paper
OJ8578	*lite-1(ce314) gur-3(ok2245) unc-9(e101) X; vjEx2548*	Fig. 4d-f, Fig. S6b	This paper
OJ8615	*frpr-21(tm4669) II; lite-1(ce314) gur-3(ok2245) X; vjEx2548*	Fig. 5b, c, Fig. S8a-c	This paper
OJ8635	*frpr-21(tm4669) II; lite-1(ce314) gur-3(ok2245) frpr-17(vj265) X; vjEx2548*	Fig. 5b, c, Fig. S8c	This paper
OJ8740	*frpr-21(tm4669) II; lite-1(ce314) gur-3(ok2245) frpr-17(vj265) X; vjEx2548; vjEx2838 [pUC276 (Pnmur-3(Δ)::frpr-21 cDNA) 25 ng/μL]*	Fig. 5b	This paper
OJ8976	*flp-9(yn36) IV; lite-1(ce314) gur-3(ok2245) frpr-17(vj265) X; vjEx2548*	Fig. 5b, c, Fig. S8c	This paper
OJ9653	*flp-9(yn36) IV; lite-1(ce314) gur-3(ok2245) frpr-17(vj265) X; vjEx2548; vjEx3128 [pMH189 (Pflp-9::flp-9 cDNA) 25 ng/μL]*	Fig. 5b	This paper
OJ9652	*flp-9(yn36) IV; lite-1(ce314) gur-3(ok2245) frpr-17(vj265) X; vjEx2548; vjEx2867 [pMH351 (Punc-47::flp-9 cDNA) 25 ng/μL]*	Fig. 5b	This paper
OJ8977	*frpr-21(tm4669) II; flp-9(yn36) IV; lite-1(ce314) gur-3(ok2245) frpr-17(vj265) X; vjEx2548*	Fig. 5b, Fig. S8c	This paper
OJ8875	*vjEx2899 [pUC281 (Pnmur-3(Δ)::frpr-21 cDNA::GFP) 25 ng/μL]*	Fig. 5d	This paper
OJ5929	*vjEx1785 [pMH351 (Punc-47::flp-9 cDNA) 25 ng/μL]*	Fig. 5e	This paper
OJ6572	*frpr-21(tm4669) II; vjEx1785 [pMH351 (Punc-47::flp-9 cDNA) 25 ng/μL]*	Fig. 5e	This paper
OJ8975	*flp-9(yn36) IV; lite-1(ce314) gur-3(ok2245) X; vjEx2548*	Fig. 5c, f, Fig. S8a-c	This paper
OJ8856	*lite-1(ce314) gur-3(ok2245) X; vjEx2548; vjEx2867 [pMH351 (Punc-47::flp-9 cDNA) 25 ng/μL]*	Fig. 5f, Fig. S8a, b	This paper
OJ8857	*frpr-21(tm4669) II; lite-1(ce314) gur-3(ok2245) X; vjEx2548; vjEx2867 [pMH351 (Punc-47::flp-9 cDNA) 25 ng/μL]*	Fig. 5f	This paper
OJ9400	*lite-1(ce314) gur-3(ok2245) X; vjEx2548; vjEx2838 [pUC276 (Pnmur-3(Δ)::frpr-21 cDNA) 25 ng/μL]*	Fig. 5f	This paper
OJ9017	*frpr-21(tm4669) II; lite-1(ce314) gur-3(ok2245) X; vjEx2548; vjEx2838 [pUC276 (Pnmur-3(Δ)::frpr-21 cDNA) 25 ng/μL]*	Fig. 5f	This paper
OJ8202	*lite-1(ce314) gur-3(ok2245) nmur-3(vj353) X; vjEx2548*	Fig. S7b, c	This paper
OJ8579	*lite-1(ce314) gur-3(ok2245) nmur-3(vj353) frpr-17(vj265) X; vjEx2548*	Fig. S7b	This paper
OJ8617	*srd-32(vj308) V; lite-1(ce314) gur-3(ok2245) X; vjEx2548*	Fig. S7b, c	This paper
OJ9403	*srd-32(vj308) V; lite-1(ce314) gur-3(ok2245) frpr-17(vj265) X; vjEx2548*	Fig. S7b	This paper
OJ8618	*npr-23(vj298) I; lite-1(ce314) gur-3(ok2245) X; vjEx2548*	Fig. S7b, c	This paper
OJ8637	*npr-23(vj298) I; lite-1(ce314) gur-3(ok2245) frpr-17(vj265) X; vjEx2548*	Fig. S7b	This paper
OJ8616	*T11F9.1(ok2284) V; lite-1(ce314) gur-3(ok2245) X; vjEx2548*	Fig. S7b, c	This paper

OJ8636	*T11F9.1(ok2284) V; lite-1(ce314) gur-3(ok2245) frpr-17(vj265) X; vjEx2548*	Fig. S7b	This paper
OJ8638	*frpr-4(ok2376) II; lite-1(ce314) gur-3(ok2245) X; vjEx2548*	Fig. S7b, c	This paper
OJ8639	*frpr-4(ok2376) II; lite-1(ce314) gur-3(ok2245) frpr-17(vj265) X; vjEx2548*	Fig. S7b	This paper
OJ9615	*unc-25(e156) III; lite-1(ce314) gur-3(ok2245) X; vjEx2548*	Fig. S7d	This paper
OJ9616	*unc-25(e156) III; lite-1(ce314) gur-3(ok2245) frpr-17(vj265) X; vjEx2548*	Fig. S7d	This paper
OJ9617	*dmsr-1(sy1522) V; lite-1(ce314) gur-3(ok2245) X; vjEx2548*	Fig. S8d	This paper
OJ9618	*dmsr-1(sy1522) V; lite-1(ce314) gur-3(ok2245) frpr-17(vj265) X; vjEx2548*	Fig. S8d	This paper
OJ9619	*dmsr-7(sy1539) V; lite-1(ce314) gur-3(ok2245) X; vjEx2548*	Fig. S8d	This paper
OJ9620	*dmsr-7(sy1539) V; lite-1(ce314) gur-3(ok2245) frpr-17(vj265) X; vjEx2548*	Fig. S8d	This paper
OJ9586	*egl-6(n4537) X; vjEx2548*	Fig. S8d	This paper
OJ9592	*egl-6(n4537) frpr-17(vj265) X; vjEx2548*	Fig. S8d	This paper
OJ9622	*frpr-8(sy1362) X; vjEx2548*	Fig. S8d	This paper
OJ9629	*frpr-8(sy1362) frpr-17(vj265) X; vjEx2548*	Fig. S8d	This paper

Supplementary Table 2: Oligonucleotides used in this study

Sequence		Oligos	Source
nmur-3(3kb) promoter	forward	ccccccGCATGCctgtttcaagattcgggaca	[3]
	reverse	ccccccCCCGGGATCCggcttcaattagttgtgtca	
nmur-3(1kb) promoter	forward	ccccccGCATGCgcgtgagcaaaatctatgttg	This paper
	reverse	ccccccCCCGGGATCCggcttcaattagttgtgtca	
nmur-3(Δ) promoter	forward	ccccccGCATGCctgtttcaagattcgggaca	This paper
	reverse	ccccccCCCGGGATCCcaacgagttgaacgtgtgtt	
arg-1 promoter	forward	ccccGCATGCaagagtttaagacgtcgca	This paper
	reverse	ccccGGATCCctttaatgatgtctagtag	
egl-18 basal promoter	forward	ccccccGGATCCctccatagtagtacattttaaggt	[3]
	reverse	ccccccCCCGGGatagactgtgtggagacac	
flp-9 promoter	forward	ccccccGCATGCgcatgatgagaacgaatttaatc	This paper
	reverse	ccccccGGATCCttttttcttctttgaaacaaaaaatg	
ICE	forward	CCCCCCgctagcAAAAatggccgacaaggtcctg	This paper
	reverse	CCCCCCggtaccttaatgtcctgggaagaggtag	
TeTx	forward	CCCCCCGCTAGCAAAAatgccgatcaccatcaacaac	This paper
	reverse	ccccccGCGGCCGCttaagcggtacggttgtacagg	
flp-22 cDNA	forward	CCCCCCgctagcAAAAATGAACCGTTCCATGATTG	This paper
	reverse	ccccGGTACCttaATAATCCTGTTCAGAAACTG	
frpr-17 cDNA	forward	ccccgctagcaaaaATGGATGATTCCGTGGATATTTATG	This paper
	reverse	ccccggtaccTCAAATAAGTACCTCTGGTTG	
PH domain::miniSOG	forward	ccccccgctagcaaaaATGGACTCGGGTAGGGACTTCC	This paper
	reverse	ccccccGCGGCCGCttatccggaagatcctccatc	
SL2::mCherry	forward	CCCCCCgcggccgcGCTGTCTCATCCTACTTTCAC	This paper
	reverse	CCCCCggtaccTTACTTGTACAGCTCGTCCA	
frpr-21 cDNA	forward	ccccGCTAGCaaaaATGGATCAAATAACAAGCAC	This paper
	reverse	ccccGGTACCTTACGAGCTGTTCGTCTCG	
flp-9 cDNA	forward	ccccGCTAGCaaaaATGAATCAATTTTATGC	This paper
	reverse	ccccGGTACCctaCTTTCTTCCAAATCGAAC	
**For 3' terminal fusion with *Venus*, *GFP*, *mCherry*, or *linker::mTurquoise2***			
wdr-23b NLS	forward	CTAGCAAAAatgccttacaaaagacattcctcttcaaatctgaaaaggA	This paper
	reverse	CCGGTccttttcagatttgaagaggaatgtcttttgtaaggcatTTTTG	
frpr-17 cDNA	forward	ccccgctagcaaaaATGGATGATTCCGTGGATATTTATG	This paper
	reverse	ccccaccggtAATAAGTACCTCTGGTTGAG	
unc-9 cDNA	forward	CCCCCCgctagcaaaaATGAGTATGCTATTGTATTATTTCGCG	This paper
	reverse	CCCCCCaccggtCACGTCGTGCATTTTTCCTTC	
frpr-21 cDNA	forward	ccccGCTAGCaaaaATGGATCAAATAACAAGCAC	This paper
	reverse	ccccACCGGTCGAGCTGTTCGTCTCGATG	
**For *flp-22 cDNA::pHluorin::flp-22 cDNA***			
flp-22 5' fragment	forward	CCCCCCgctagcAAAAATGAACCGTTCCATGATTG	This paper
	reverse	GACGGGGAcctaggcttACCGGTACCGAATCGCATCCATTTGG	
flp-22 3' fragment	forward	GATTCGGTACCGGTaagcctaggTCCCCGTCAGCCAAATGG	This paper
	reverse	ccccGGTACCttaATAATCCTGTTCAGAAACTG	
pHluorin	forward	ccccccACCGGTAGTAAAGGAGAAGAACTTTTC	This paper
	reverse	CCCCCCcctaggTTTGTATAGTTCATCCATGCC	

Supplementary References

1 Mathies, L. D. *et al.* mRNA profiling reveals significant transcriptional differences between a multipotent progenitor and its differentiated sister. *BMC Genomics* **20**, 427, doi:10.1186/s12864-019-5821-z (2019).

2 Wang, H. *et al.* Neuropeptide secreted from a pacemaker activates neurons to control a rhythmic behavior. *Curr Biol* **23**, 746-754, doi:10.1016/j.cub.2013.03.049 (2013).

3 Choi, U., Wang, H., Hu, M., Kim, S. & Sieburth, D. Presynaptic coupling by electrical synapses coordinates a rhythmic behavior by synchronizing the activities of a neuron pair. *Proc Natl Acad Sci U S A* **118**, doi:10.1073/pnas.2022599118 (2021).
