# Supplementary material for: The head mesodermal cell couples FMRFamide neuropeptide signaling with rhythmic muscle contraction in C. elegans
Source: Nat Commun. 2023 Jul 14;14:4218. doi: 10.1038/s41467-023-39955-8 (PMC10349088; doi:10.1038/s41467-023-39955-8)
Supplement: Supplementary file 3 — Description of Additional Supplementary Files [file 41467_2023_39955_MOESM3_ESM.pdf]

<u>Description of Additional Supplementary Files</u>

Supplementary Movie 1. Calcium imaging of a freely moving wild-type day 1 adult co-expressing GCaMP6 in AVL and hmc (under the *nmur-3* promoter) and GCaMP3 in intestine (under the *nlp-40* promoter). Movie shows one cycle of the DMP in which a calcium spike in the AVL cell body (including the axon) and in the hmc cell body (including the processes) were observed at the same time (within 250ms of the calcium spike initiation in AVL) about 3 seconds after the intestinal calcium oscillation.

Supplementary Movie 2. Calcium imaging of a freely moving *frpr-17* mutant day 1 adult co-expressing GCaMP6 in AVL and hmc (under the *nmur-3* promoter) and GCaMP3 in intestine (under the *nlp-40* promoter). Movie shows one cycle of the DMP in which a calcium spike only occurred in the AVL cell body (including the axon) but not in hmc about 3 seconds after the intestinal calcium oscillation.

Supplementary Movie 3. Calcium imaging of a freely moving *unc-9* mutant day 1 adult co-expressing GCaMP6 in AVL and hmc (under the *nmur-3* promoter) and GCaMP3 in intestine (under the *nlp-40* promoter). Movie shows one cycle of the DMP in which a calcium spike occurred in the AVL cell body (including the axon) and in the hmc cell body (including the processes) were observed at the same time (within 250ms of the calcium spike initiation in AVL) about 3 seconds after the intestinal calcium oscillation but not followed by aBoc.

Supplementary Movie 4. Calcium imaging of a freely moving *unc-54* mutant day 1 adult co-expressing GCaMP6 in AVL and hmc (under the *nmur-3* promoter) and GCaMP3 in intestine (under the *nlp-40* promoter). Movie shows one cycle of the DMP in which a calcium spike occurred in the AVL cell body (including the axon) and in the hmc cell body (including the processes) were observed at the same time (within 250ms of the calcium spike initiation in AVL) about 3 seconds after the intestinal calcium oscillation but not followed by aBoc.
