# Supplementary material for: The head mesodermal cell couples FMRFamide neuropeptide signaling with rhythmic muscle contraction in C. elegans
Source: Nat Commun. 2023 Jul 14;14:4218. doi: 10.1038/s41467-023-39955-8 (PMC10349088; doi:10.1038/s41467-023-39955-8)
Supplement: Supplementary file 8 — Reporting Summary [file 41467_2023_39955_MOESM8_ESM.pdf]

nature portfolio

Corresponding author(s): Derek Sieburth
Last updated by author(s): Jun 5, 2023

Reporting Summary

Nature Portfolio wishes to improve the reproducibility of the work that we publish. This form provides structure for consistency and transparency in reporting. For further information on Nature Portfolio policies, see our Editorial Policies and the Editorial Policy Checklist.

Statistics

For all statistical analyses, confirm that the following items are present in the figure legend, table legend, main text, or Methods section.

n/a	Confirmed	
☐	☒	The exact sample size (*n*) for each experimental group/condition, given as a discrete number and unit of measurement
☐	☒	A statement on whether measurements were taken from distinct samples or whether the same sample was measured repeatedly
☐	☒	The statistical test(s) used AND whether they are one- or two-sided<br>*Only common tests should be described solely by name; describe more complex techniques in the Methods section.*
☒	☐	A description of all covariates tested
☐	☒	A description of any assumptions or corrections, such as tests of normality and adjustment for multiple comparisons
☐	☒	A full description of the statistical parameters including central tendency (e.g. means) or other basic estimates (e.g. regression coefficient) AND variation (e.g. standard deviation) or associated estimates of uncertainty (e.g. confidence intervals)
☐	☒	For null hypothesis testing, the test statistic (e.g. *F*, *t*, *r*) with confidence intervals, effect sizes, degrees of freedom and *P* value noted<br>*Give P values as exact values whenever suitable.*
☒	☐	For Bayesian analysis, information on the choice of priors and Markov chain Monte Carlo settings
☒	☐	For hierarchical and complex designs, identification of the appropriate level for tests and full reporting of outcomes
☒	☐	Estimates of effect sizes (e.g. Cohen's *d*, Pearson's *r*), indicating how they were calculated

Our web collection on statistics for biologists contains articles on many of the points above.

Software and code

Policy information about availability of computer code

Data collection	Metamorph 7.0, Etho software
Data analysis	ImageJ 1.53, GraphPad Prism 9, Microsoft Excel

For manuscripts utilizing custom algorithms or software that are central to the research but not yet described in published literature, software must be made available to editors and reviewers. We strongly encourage code deposition in a community repository (e.g. GitHub). See the Nature Portfolio guidelines for submitting code & software for further information.

Data

Policy information about availability of data

All manuscripts must include a data availability statement. This statement should provide the following information, where applicable:

- Accession codes, unique identifiers, or web links for publicly available datasets
- A description of any restrictions on data availability
- For clinical datasets or third party data, please ensure that the statement adheres to our policy

All data that support the findings of this study are available within the paper and its supplementary files. Source data are provided with this paper. The source data can also be accessed at figshare. DOI: https://doi.org/10.6084/m9.figshare.22756898.

Research involving human participants, their data, or biological material

Policy information about studies with human participants or human data. See also policy information about sex, gender (identity/presentation), and sexual orientation and race, ethnicity and racism.

Reporting on sex and gender	Not applicable
Reporting on race, ethnicity, or other socially relevant groupings	Not applicable
Population characteristics	Not applicable
Recruitment	Not applicable
Ethics oversight	Not applicable

Note that full information on the approval of the study protocol must also be provided in the manuscript.

Field-specific reporting

Please select the one below that is the best fit for your research. If you are not sure, read the appropriate sections before making your selection.

☒ Life sciences ☐ Behavioural & social sciences ☐ Ecological, evolutionary & environmental sciences

For a reference copy of the document with all sections, see nature.com/documents/nr-reporting-summary-flat.pdf

Life sciences study design

All studies must disclose on these points even when the disclosure is negative.

Sample size	For all behavioral and imaging assays between 3 and 21 animals of a given genotype were analyzed. No data were excluded from the analysis
Data exclusions	none
Replication	At least three biological replicates were performed for each experiment. All data was replicated successfully.
Randomization	We randomly selected individuals of each genotype for phenotypic analysis.
Blinding	For fluorescence calcium imaging, blinding was not necessary since the data collection of fluorescence time lapse images was done prior to data analysis. The changes in fluorescence intensity were too subtle to be noticed during imaging. Therefore, no bias was introduced during data collection. During data analysis, blinding was not necessary for the two following reasons. First, for quantifying calcium spike frequencies, a strict criterion was applied where only events showing more than a 50% change in $\Delta F/F0$ were considered as spike events, while those below 50% were considered as no spike events. Second, for quantifying the calcium spike intensities to obtain data of peak amplitude, rise time, and half-decay time, a clear first cycle from each animal was analyzed.<br>For aBoc assays, the contractions are reported as either occurring or not occurring (a binary readout), and the contractions are easily detectable, thereby limiting bias in these assays.

Reporting for specific materials, systems and methods

We require information from authors about some types of materials, experimental systems and methods used in many studies. Here, indicate whether each material, system or method listed is relevant to your study. If you are not sure if a list item applies to your research, read the appropriate section before selecting a response.

Materials & experimental systems

n/a	Involved in the study	
☒	☐	Antibodies
☒	☐	Eukaryotic cell lines
☒	☐	Palaeontology and archaeology
☐	☒	Animals and other organisms
☒	☐	Clinical data
☒	☐	Dual use research of concern
☒	☐	Plants

Methods

n/a	Involved in the study	
☒	☐	ChIP-seq
☒	☐	Flow cytometry
☒	☐	MRI-based neuroimaging

Animals and other research organisms

Policy information about studies involving animals; ARRIVE guidelines recommended for reporting animal research, and Sex and Gender in Research

Laboratory animals	One day hermaphrodite adults of C. elegans
Wild animals	none
Reporting on sex	hermaphrodites only
Field-collected samples	none
Ethics oversight	The study did not require an ethical approval

Note that full information on the approval of the study protocol must also be provided in the manuscript.
